# Multiple interaction modes between saccharin and sweet taste receptors determine a species‐dependent response to saccharin

**DOI:** 10.1002/2211-5463.13355

**Published:** 2021-12-31

**Authors:** Xiangzhong Zhao, Meng Liu, Meng Cui, Bo Liu

**Affiliations:** ^1^ Department of Food Science and Engineering Qilu University of Technology Jinan China; ^2^ Department of Pharmaceutical Sciences Northeastern University Boston MA USA

**Keywords:** allosteric binding site, inhibition, interaction modes, saccharin, species‐dependent sweet taste, sweet taste receptor

## Abstract

Saccharin is a commonly used artificial sweetener that exhibits both sweetening and sweet inhibition activities. The species‐dependent response towards saccharin and the interaction between saccharin and the sweet taste receptor T1R2/T1R3 remain elusive. In this study we used mismatched chimeras of T1R2 and T1R3 and calcium mobilization functional analysis to reveal a detailed species‐dependent response towards saccharin of human, squirrel monkey, and mouse sweet taste receptors. Our findings, combined with previous results by others, suggest multiple and complex interaction modes between saccharin and the sweet taste receptor, which are helpful guidelines for effective modulation of the sweet taste by sweetener/sweet inhibitors.

## Graphical abstract

Saccharin exhibits both sweetening and sweet inhibition activities. Besides the orthosteric binding site in T1R2 for activating the human sweet taste receptor T1R2/T1R3, it appears that there are at least two allosteric inhibitory sites located in T1R2 and T1R3 respectively to inhibit the receptor activity, suggesting multiple and complex interaction modes between saccharin and the heterodimeric sweet taste receptor T1R2/T1R3.

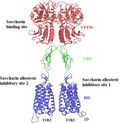

**Abbreviations**
CRD: cysteine‐rich domain
GPCR: G protein‐coupled receptor
HD: heptahelical domain
ID: intracellular domain
SEM: standard error of mean
TM: transmembrane
VFTM: Venus flytrap module

It has been well‐known that the sweet taste perception in mammalians is broadly mediated by the sweet taste receptor, which belongs to the family C G protein‐coupled receptors (GPCRs) [1]. The receptor is preferentially expressed at the membrane of tastebuds cells on the tongue, and consists of two monomers, T1R2 and T1R3, which are linked by the noncovalent bond interactions [2]. Various sweeteners, natural sugars, sweet amino acids, artificial sweeteners, and sweet‐tasting proteins can bind and interact with the receptor, trigger the conformational shift from rest to the active state, activate the coupled G protein, induce the downstream signal transduction, and then produce the sweet taste perception [3].

The heterodimeric sweet taste receptor T1R2/T1R3 is composed of three domains: a large extracellular domain, which includes a Venus flytrap module (VFTM) and a cysteine‐rich domain (CRD), a heptahelical domain (HD), which includes seven transmembrane helices (TMs 1–7), and an intracellular domain (ID), which interacts with the G protein (Fig. 1) [4]. It has been revealed that there are multiple binding sites in the sweet taste receptor for its interaction with various sweet compounds, which are meaningful for understanding and modifying the properties of sweeteners and sweet modulators [5].

**Fig. 1:**
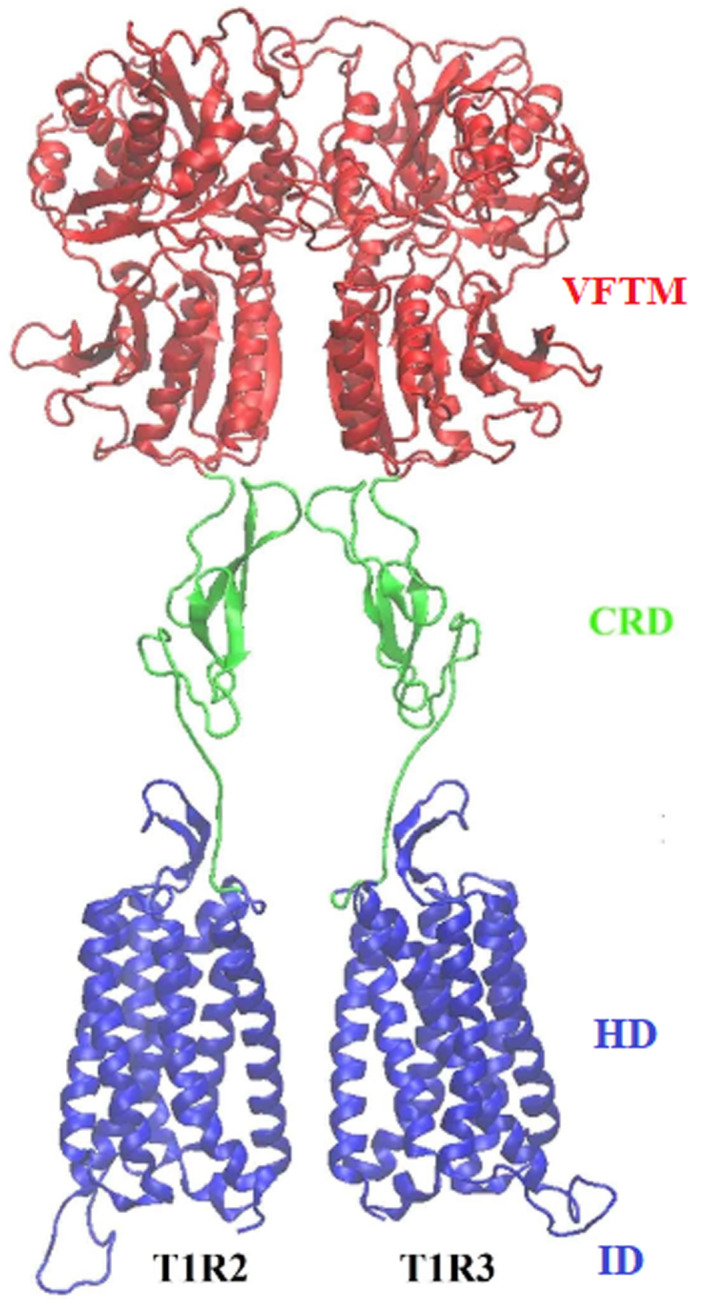
Schematic representation of the overall modeled structure of the human sweet taste receptor T1R2/T1R3. The VFTM, CRD, and HD+ID domains are colored in red, light green, and blue, respectively. This figure was made with pymol software.

The artificial sweetener saccharin has been shown to elicit both human sweet sensation (< 3 mm) and sweet inhibitory (> 3 mm) responses, depending on the concentration of stimulation. Based on the species‐dependent responses towards Na‐saccharin and functional analysis of the human/rat chimeras of sweet taste receptor T1R2/T1R3, it has been suggested that the human T1R3 is the allosteric binding site for inhibition by Na‐saccharin [6]. However, Masuda *et al*. reported that the extracellular VFTM region of human T1R2 is an orthosteric binding site for the sweetness of saccharin [7], indicating multiple interaction sites between saccharin and the sweet taste receptor. Nevertheless, the detailed species‐dependent response towards saccharin remains unclear until now.

In this study, based on functional analysis of the species‐dependent responses towards saccharin, we reveal a new interactive profile between saccharin and the sweet taste receptor T1R2/T1R3.

## Materials and methods

### Materials

D‐tryptophan (product number T9753, purity ≥98%) was purchased from Sigma‐Aldrich (St. Louis, MO, USA). Sucralose (product number 29329990.99, purity ≈99.2%) was obtained from Tongyuan Sweetener Co. Ltd (Huanggang, China). Stevioside (purity ≈90%) was obtained from NuSci Institute & Corp (Walnut, CA, USA). Saccharin (product number 47839, purity ≈99.9%) was obtained from Supelco (Bellefonte, PA, USA). All other chemicals and reagents were analytical grade and were from Invitrogen (La Jolla, CA, USA).

### Constructs and molecular simulations

The human, squirrel monkey, and mouse T1R2s and T1R3s (mouse C57BL/6 strain) constructs were generated with the expression vector pcDNA3.1 (Invitrogen) as described previously [8]. The Gα16‐gust44 construct was generated with the vector pcDNA3.1, as described previously [9].

The homology model of full‐length human T1R2/T1R3 was constructed using the Swiss‐Model program (http://swissmodel.expasy.org/) with the heterodimeric human metabotropic GABA(B) receptor (PDB: 6UO8) as the template. A sequence alignment of the human T1R2 and T1R3 and template sequences was carried out with the ClustalW program [10], and the human T1R2 and T1R3 were uploaded as hetero targets for modeling, respectively. The resulting model was evaluated with the Verify 3D program and the top scored model was selected [11].

### Cell‐based function assay

The calcium mobilization assay of the responses of sweet taste receptors and chimeras towards various sweeteners was performed as our previously described procedures [12]. Briefly, the T1R2/3 and Gα16‐gust44 constructs were cotransfected into the HEK293E cells and then expressed for about 48 h. Calcium mobilization was obtained with a fluorescence change (excitation, 488 nm; emission, 525 nm; cutoff, 515 nm) on a FlexStation 3 system (Molecular Devices, Palo Alto, CA, ISA) after stimulation with 2 × tastants. The responses were represented as the percentage of change (peak fluorescence‐baseline fluorescence level, denoted ΔF) from its baseline fluorescence level (denoted F). Each experiment was conducted in triplicate and the results were averaged [12].

## Results

### Species‐dependent response to saccharin

**Fig. 2:**
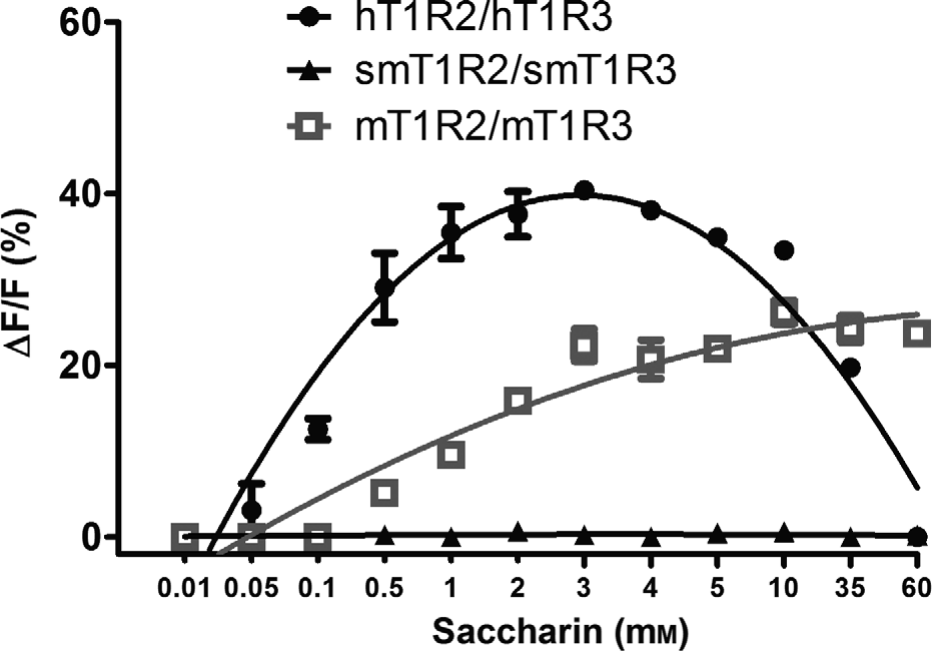
Dose‐dependent activation and inhibition of human, squirrel monkey, and mouse sweet taste receptors by saccharin. The figure shows the dose–response curve of human, squirrel monkey, and mouse sweet taste receptors (h, sm, and mT1R2/T1R3s) towards saccharin. Each assay was performed in three independent replicates, and the data were analyzed using the graphpad Prism software (San Diego, CA, USA) and shown in average with SEM (standard error of mean).

The constructs of sweet taste receptors and Gα16‐gust44 were transfected into the HEK293E cells, and their expression has been demonstrated in our and other previous studies [12, 13, 14]. Subsequently, the responses to saccharin of human, squirrel monkey, and mouse sweet taste receptors were investigated with the calcium mobilization assay. As shown in Fig. 2, squirrel monkey sweet receptor could not respond to saccharin. The human receptor responded to saccharin at low concentrations (< 3 mm), but the responses were inhibited when the concentration of saccharin was above 3 mm. However, mouse receptor showed a dose‐dependent increase of response to saccharin and no inhibition was found at the concentrations of saccharin tested up to 60 mm. These results demonstrated the species‐dependent responses to saccharin for human and mouse but not squirrel monkey sweet taste receptors, while species‐specific inhibition of the response by saccharin at high concentrations (> 3 mm) for human but not mouse receptors.

### Saccharin inhibits the human and squirrel monkey sweet taste but not that of the mouse

**Fig. 3:**
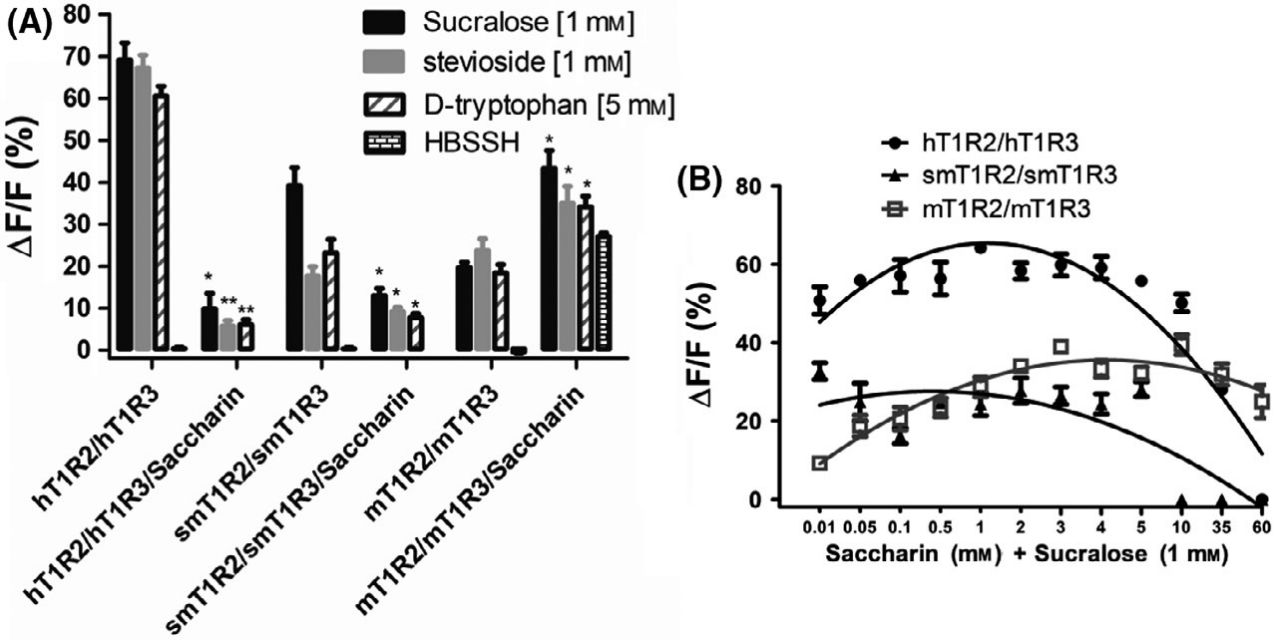
Activation/inhibition of the responses of human, squirrel monkey, and mouse sweet taste receptors by saccharin. (A) Saccharin (60 mm) inhibited the response of human and squirrel monkey but not mouse sweet taste receptors towards sucralose, stevioside, and d‐tryptophan, respectively. The asterisks indicate significant differences tested by the two‐tailed paired *t* test (statistical significance *P < 0.05; **P < 0.01) compared with the response of receptors without saccharin added. The Hank's Balanced Salt Solution HEPES (HBSSH) buffer was used as the blank. Note that the bars for smT1R2/smT1R3/Saccharin and hT1R2/hT1R3/Saccharin (HBSSH bar) were not visible due to no or little responses of the smT1R2/smT1R3 and hT1R2/hT1R3 to saccharin (60 mm), as illustrated in Fig. 2. (B) Dose–response curve for inhibition of the response of human and squirrel monkey but not mouse sweet taste receptors towards sucralose (1 mm) by saccharin. Each assay was performed in three independent replicates, and the data were analyzed using the graphpad Prism software and shown in average with SEM (standard error of mean).

To further determine the inhibitory property of saccharin, we examined the species‐dependent response to three representative sweeteners in the presence of 60 mm saccharin. We selected three sweeteners: sucralose, stevioside, and d‐tryptophan, which can be perceived by all three species. As shown in Fig. 3A, the responses of human and squirrel monkey T1R2/T1R3 to sucralose, stevioside, and D‐tryptophan were intensively inhibited by 60 mm saccharin, whereas the response of mouse T1R2/T1R3 could not be inhibited. The dose–response experiment showed the inhibitory efficiency of the response towards sucralose by saccharin in human, squirrel monkey, and mouse (Fig. 3B). It is interesting that the mouse responds to saccharin as a sweetener rather than an inhibitor, while the squirrel monkey responds to saccharin as an inhibitor rather than a sweetener.

### T1R2 subunit determines the species‐dependent inhibitory sensitivity to saccharin

**Fig. 4:**
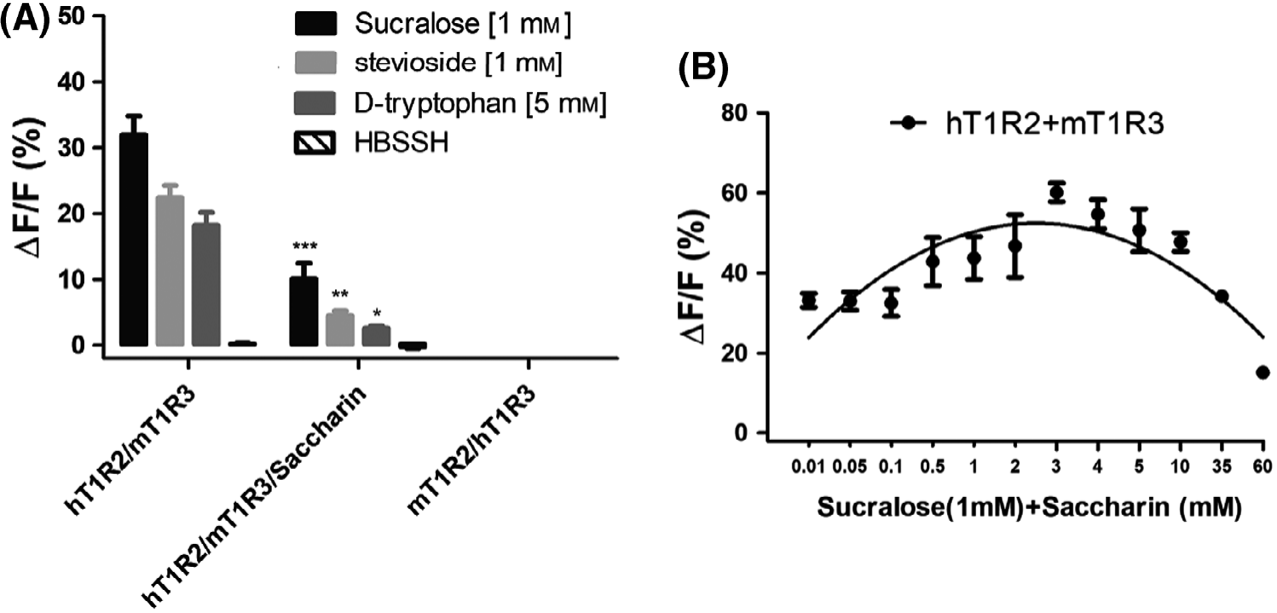
Inhibition of the responses of chimeric human and mouse T1R2/T1R3 receptors by saccharin. (A) Responses of hT1R2/mT1R3 and mT1R2/hT1R3 towards sucralose, stevioside, and d‐tryptophan and their inhibition by 60 mm saccharin. The asterisks indicate significant differences tested by the two‐tailed paired *t* test (*P < 0.05; **P < 0.01; ***P < 0.001) compared with the response of receptors without saccharin added. The HBSSH buffer was used as the blank. (B) Dose–response curve for inhibition of the responses of hT1R2/mT1R3 towards sucralose (1 mm) by saccharin. Each assay was performed in three independent replicates, and the data were analyzed using the graphpad Prism software and shown in average with SEM (standard error of mean).

Using mismatched chimeric human/squirrel monkey T1R2/3, we have previously shown that human T1R2 is a potential binding site of the receptor for saccharin [8], and Masuda et al. demonstrated that saccharin binds into the extracellular VFTM region of human T1R2 for eliciting its sweetness [7]. To further investigate the molecular determinant that is responsible for the inhibitory sensitivity towards saccharin, we examined the response of the mismatched chimeric human, squirrel monkey, and mouse T1R2 and T1R3 (namely h, sm, mT1R2/3, respectively). Because saccharin inhibits sweet taste in humans and squirrel monkey but not mouse (Fig. 3), we used the chimeric human/mouse T1R2/3 for saccharin inhibitory sensitivity. Consistent with the previously reported results, we found that the chimeric mouse T1R2 with human T1R3 (mT1R2/hT1R3) showed no response to any sweeteners tested, implying that this chimeric receptor could not be efficiently expressed or targeted on the membrane [13]. However, replacement of mouse T1R2 with human T1R2 (hT1R2/mT1R3) led to a gain of the inhibitory sensitivity to saccharin (Fig. 4A,B). These results suggest that residues in human T1R2 mediate the species‐dependent inhibitory sensitivity to saccharin between humans and mouse.

## Discussion

In this study we reported the detailed species‐dependent responses towards saccharin of human, squirrel monkey, and mouse sweet taste receptors both for sweetening and inhibitory activities, and revealed that the sweet taste receptor T1R2 is a novel allosteric binding site for inhibition by saccharin. As shown in Figs. 3 and 4, saccharin at high concentrations (> 3 mm) can inhibit the response of human but not mouse sweet taste receptor T1R2/T1R3 towards sucralose, stevioside, and D‐tryptophan, while the response of mismatched human T1R2 and mouse T1R3 (replacement of mouse T1R2 by human T1R2) towards these sweeteners can be inhibited by saccharin (> 3 mm). These results indicate that saccharin at high concentrations can bind at one or more allosteric sites located in human T1R2 to inhibit the response of human sweet taste receptor towards various sweeteners. However, it was previously described that the human T1R3 is an allosteric binding site of saccharin for inhibiting sweet taste [6]. Moreover, based on the species‐dependent sweet taste towards saccharin between humans and squirrel monkey, we and other researchers have shown that the extracellular VFTM region of human T1R2 is a binding site of saccharin for eliciting its sweetness [7, 8]. Therefore, it appears that at least three binding sites (two allosteric sites in T1R2 and T1R3, respectively, for inhibitory activity and one site in T1R2 for sweetening activity) in the human sweet taste receptor for saccharin sensitivity, and multiple interaction modes may exist between saccharin and the sweet taste receptor.

A two‐state allosteric model was proposed to explain the sweet “water‐taste” that removing the sweet taste inhibitors such as saccharin, acesulfame‐K, and lactisole located at the allosteric binding sites shifts the receptor from a high‐energy inhibited state to the constitutively active state, thus stimulating the sweetness upon water rinsing [6]. Our present result demonstrates a more detailed dose‐based species‐dependent taste towards saccharin, and extends this model with the finding that saccharin could bind into an allosteric inhibitory site located in T1R2. At low concentrations (<3 mm), Na‐saccharin preferentially binds into the high‐affinity orthosteric site in T1R2, activating the receptor. When the concentrations increase (>3 mm), the excessive saccharin molecules can bind to allosteric inhibitory sites located in human T1R2, and when more saccharin is added, they can probably bind with both T1R2 and T1R3, giving rise to the inhibited state of the receptor. Interestingly, we have reported that the HD of human T1R2 is an allosteric binding site for the sweet inhibitor amiloride [15]. It is meaningful to further investigate that the dose‐dependent discrepancy or preference of binding between T1R2 and T1R3 and the crosstalk between the two monomers of sweet taste receptor upon saccharin activation/inhibition, which should be helpful guidance for rational design of effective sweetener/sweet taste modulators.

## Conflict of interest

The authors declare no conflict of interest.

## Data accessibility

The data are available from the corresponding author upon reasonable request.

## Author contributions

MC and BL conceived and designed the project, XZ and ML acquired the data, MC and BL analyzed and interpreted the data, and BL and MC wrote the article.
